# Laparoscopic Repair of Spontaneous Esophageal Perforation After Multiple Endoscopic Failures

**DOI:** 10.7759/cureus.26784

**Published:** 2022-07-12

**Authors:** Murugappan Nachiappan, Ravikiran Thota, Srikanth Gadiyaram

**Affiliations:** 1 Department of Surgical Gastroenterology and Minimally Invasive Surgery, Sahasra Hospitals, Bengaluru, IND

**Keywords:** over-the-scope clips, case report, endotherapy, laparoscopy, sems, spontaneous esophageal perforation, boerhaave syndrome

## Abstract

Spontaneous esophageal perforation (SEP) (Boerhaave syndrome) carries high morbidity and mortality. Delay in diagnosis, because of the non-specific complaints and the rarity of the condition, further increases the mortality. While patients diagnosed early can be managed by primary closure of esophageal perforation, those presenting beyond 24 hours often require an esophagectomy with salivary diversion and feeding access with a plan for the reconstruction of the alimentary tract at a later date. In a minority of patients with a controlled esophageal fistula and feeding access, source control could be achieved by endotherapy. Patients with mediastinitis and associated systemic sepsis would be better served by surgical intervention. We present a case of an SEP with a delayed diagnosis, who underwent three unsuccessful endotherapy attempts and decortication before referral for surgical repair. The patient had an established esophageal fistula. He underwent a laparoscopic repair of the fistula. Postoperative recovery was uneventful. At the one-year follow-up, the patient was asymptomatic and had gained weight. Though surgery is the treatment of choice, the optimal management of SEP with delayed diagnosis is not clearly defined. In the current era of advanced endotherapy, more cases are being managed endoscopically. However, they carry a high failure rate, resulting in increased morbidity among the patients. Early involvement of a surgical team in the decision-making is crucial for optimal outcomes of the disease.

## Introduction

Spontaneous esophageal perforation (SEP) because of barotrauma is sometimes managed as a non-esophageal disease early in the course of the illness [1]. SEP is associated with significant morbidity and mortality [2]. There are a variety of management options available to the treating physician [3,4]. Surgical intervention to close the esophageal perforation provides the best survival outcomes in patients with SEP presenting within 24 hours [3,5]. Morbidity and mortality increase in patients with delayed presentation (over 24 hours) [6]. Optimal management in late presentation is not clear. The options include conservative management, endoscopic procedures, and surgery. With the availability of advanced endoscopes and accessories, many of these are being managed with endotherapy. However, the requirement of salvage therapy, either as a second endoscopic procedure or a surgical procedure, because of failure is 25-50% for endoscopic procedures [7,8]. In a retrospective review of a small case series by Glatz et al., the failure rate was 42% in patients who were managed within 48 hours and 75% in patients who were managed with self-expandable metal stents (SEMS) after 48 hours [8]. A second attempt at stenting was unsuccessful in any patient who had failed first stenting [8]. They also necessitate the need for secondary interventions such as drainage of the pleural cavity and the mediastinum, decortication, and feeding access. Here, we report a case of Boerhaave syndrome in which the diagnosis was delayed and unsuccessfully managed with endotherapy on three occasions before referral for surgery, reiterating the above observation.

## Case presentation

**Figure 1:**
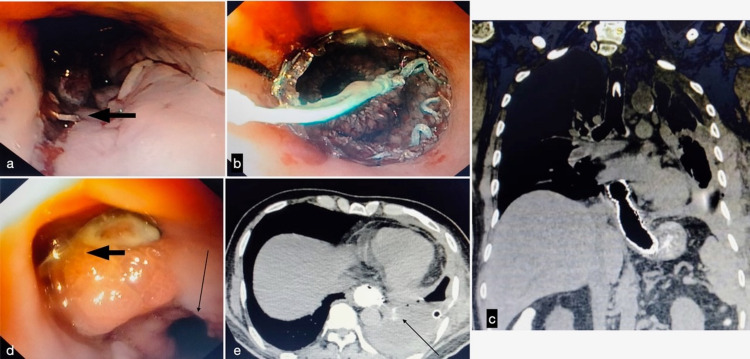
(a) Perforation closed with over-the-scope clip (block arrow). (b) SEMS in situ. (c) Stent migration. (d) Fistula (block arrow) just above the GEJ (line arrow) after removal of the SEMS. (e) Persistent contrast leak (line arrow) into the left pleural cavity after removal of the stent, also seen is a chest tube. SEMS: self-expandable metal stents; gastroesophageal junction

**Figure 2:**
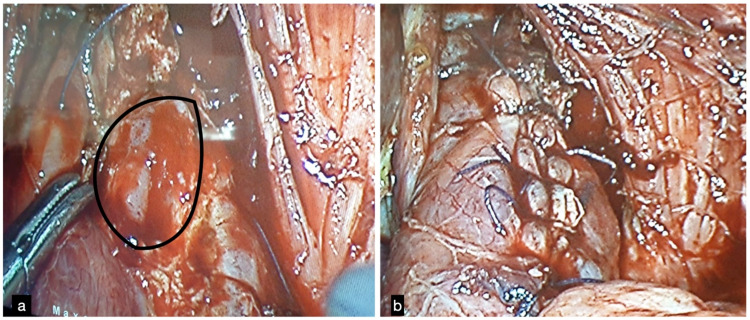
(a) Opening in the esophagus after the takedown of the fistula and refreshening of the margins (black margins). (b) Completed repair of the fistula.

A 59-year-old gentleman with no significant medical history presented with chest pain and progressive dyspnea following vomiting. In the emergency room at a local hospital, a diagnosis of lower respiratory tract infection with respiratory failure was made and managed in the intensive care unit (ICU). Progressive left pleural effusion and later hydropneumothorax necessitated a computed tomography (CT) of the chest with oral contrast, and a diagnosis of SEP was made after a five-day delay. Intercostal tube drainage was done, and with a very sick patient at hand, the treating team proceeded with an endoscopic over-the-scope (OTS) clipping in preference to surgery (Figure 1a). A contrast study done immediately showed no leak. However, the leak was evident three days later by increasing left chest purulent effusion. A feeding jejunostomy (FJ) was performed for enteral nutrition, and the patient was referred to a pulmonology unit. CT thorax with oral contrast showed a lower esophageal fistula into the left chest with empyema with collapse/consolidation. The patient underwent esophageal SEMS placement (Figure 1b). A contrast CT subsequently showed no leak, and he underwent a decortication procedure. A week after the initial placement, the SEMS had to be repositioned because of distal migration (Figures 1c, 1d). He required ICU care for the next three weeks. A CT repeated three weeks later showed a persistent leak into the left chest (Figure 1e). He was then referred to us for further management. The patient was clinically stable, had a persistent controlled low output esophageal fistula, and was receiving full enteral feeds through the feeding jejunostomy. His nutritional parameters were within normal limits. A plan for surgical intervention was made. The options considered were an esophageal exclusion with cervical esophagostomy or fistula takedown laparoscopically. An upper gastrointestinal endoscopy was performed, and the SEMS was removed. At laparoscopy, a 1.5 cm wide esophagopleural fistula was identified 3 cm above the gastroesophageal junction (GEJ) on the left, which was divided (Figure 2a). The edges were freshened and closed primarily with omental tacking (Figure 2b). A 28F tube drain was left in the mediastinum, exiting from the left anterior axillary line. Postoperatively, the patient made a rapid recovery. Nutrition was maintained through the FJ. A contrast study on day seven showed no leak, and he was started on a liquid diet graduated over the next week to a solid diet. At the one-year follow-up, he had gained weight and was asymptomatic with normal upper gastrointestinal endoscopy.

## Discussion

The principles of management of SEP with delayed diagnosis include stabilization, source control, and nutritional access. The options include conservative management, endoscopic procedures, and surgery.

Endoscopic procedures include OTS clips, SEMS, and esophageal endoscopic suturing [1,9]. Smaller perforations and those near the GEJ which prevent the safe distal landing of the SEMS have been managed with OTS clips in a few reports [9,10]. SEMS across the GEJ also increase the risk of aspirations [7]. SEMS are associated with stent migration and failure [11]. Despite the advances in endoscopic techniques, stent migration remains a concern for a fully covered stent, and anchoring sutures or clips are required [7]. Dual endotherapy where a clip is followed by SEMS placement has been used previously [9]. The requirement of salvage therapy, either as a second endoscopic procedure or a surgical procedure, because of failure is 25-50% for endoscopic procedures [7,8]. There is also a need for secondary intervention such as multiple intercostal tube placements and decortication like in our patient.

Surgical options include primary repair, T tube drainage, esophageal exclusion along with feeding access, and an esophagectomy with reconstruction in a single setting [1,12,13]. Primary repair is considered where the diagnosis is made early and minimal contamination is present, and, rarely, in delayed cases like the present one where the perforation was converted into a low-output fistula and healthy margins were available. Closure of the defect over a T tube which helps it to convert the perforation into a controlled fistula and later removal of the tube can be considered in delayed cases. Single-stage esophagectomy and reconstruction is also an option. Primary closure avoids the morbidity of the long-term drain in situ and is not a major undertaking such as an esophagectomy and should be considered where it is feasible like in our case.

The endoscopic procedures appear to be better in an iatrogenic perforation where the diagnosis is made early and minimal contamination of the pleural cavity and the mediastinum are present. It is easier to apply OTS clips in a fresh perforation compared to an inflamed, friable one [14]. The rationale for using endoscopic procedures is to negate the secondary surgical stress in a patient who is already compromised by delayed management, probably the reason multiple attempts at endoscopy were done prior to a surgical consult in our patient. However, it delayed a definitive surgical repair, which could have provided an earlier return to good health. It necessitated multiple secondary procedures such as SEMS replacement because of migration, intercostal tube placements, and decortication. The minimally invasive approach further helped to decrease postoperative pain and improved postoperative ventilation and early recovery.

Although the advances in endoscopy and stents have resulted in more patients being managed with endotherapy, it would be prudent to manage SEP with mediastinitis diagnosed within 24 hours with primary surgical repair. Patients presenting after 24 hours with mediastinitis, systemic sepsis, and hemodynamic instability should undergo surgery with salivary diversion (with or without esophagectomy) and feeding jejunostomy. In patients presenting after 24 hours, endotherapy can be considered only in a subgroup of patients who are hemodynamically stable with minimal mediastinal contamination. If one attempt at endotherapy fails, the patient must be managed surgically [8].

## Conclusions

Laparoscopic primary repair is feasible in delayed presentation of SEP with endotherapy failure and controlled esophageal fistula in a nutritionally maintained patient. The optimal management of SEP with delayed diagnosis is not clearly defined. Early involvement of a surgical team in the decision-making is crucial for optimal outcomes of the disease.
